# Acknowledgement to reviewers

**DOI:** 10.1308/003588413X13511609956930

**Published:** 2013-01

The *Annals* office would like to thank the following reviewers for kindly helping us during 2012. We greatly appreciate your time and helpful comments and hope you will continue to support us as you have done in the past. Should any of our readers wish to join the panel of reviewers, please contact the *Annals* office.

M Absar
R Ackroyd
J Adams
P Addison
A Agarwal
S Agrawal
M Akhavani
D Alderson
A Alhasso
B Alkari
R Allum
J Alonso
E Aly
S Aly
G Al-Yassari
R Anand
S Anand
I Anderson
T Andrade
J Arrowsmith
T Arulampalam
C Arun
D Back
R Baigrie
I Bailey
P Baird
C Bartram
A Basarab
S Batchelor
A Bateman
M Bater
J Beard
N Beck
M Bell
G Bentley
R Bhatia
S Bhattacharya
C Bicknell
B Birch
D Birchley
M Birks
J Blazeby
S Boddy
P Boorman
D Boyce
G Branagan
S Brearley
T Briggs
A Brightwell
D Britton
S Brown
D Brown
H Brownlow
N Brownson
J Byrne
N Campkin
N Carty
I Chakrabarti
W Chambers
K Chapple
N Chaudhuri
D Chin Song
D Clark
N Clarke
N Clay
P Clewer
C Coapes
L Condon
M Cooper
S Copeland
S Corbett
A Corder
M Corlett
P Counter
A Cowan
P Cox
D Crabbe
M Craigen
B Cresswell
G Cribb
W Cross
M Crundwell
E Cusick
R Cutress
D Dandy
I Daniels
G Datta
P Davey
H David
M Davis
E Davis
P Dawson
B De Souza
M Deakin
S Deakin
J Dearing
L Deliss
G Delves
V Deveraj
S Dhillon
T Diamond
I Donaldson
A Donne
N Downing
D Drake
D Dunlop
R Dunn
J Dunn
G Duthie
J Earnshaw
R Eckersley
C Edwards
E Efthimiou
D Elliot
A Emmanuel
S Evans
D Evriviades
K Eyres
W Faux
B Fearn
N Fiddian
P Fielding
J Fischer
A Fitzgerald-O’Connor
J Fitz-Henry
D Fleming
T Flood
L Flood
S Fullilove
D Galvin
A Gandhi
K Gardiner
S Garg
M Gargan
A Gee
P Gibbs
S Gidwani
H Giele
H Giele
J Gilbert
J Goddard
R Gompertz
M Gough
J Graham
I Grant
W Gray
A Green
T Greenwell
R Grimer
A Grobbelaar
M Grocott
R Gupta
C Gupte
J Hall
L Hamilton
C Hand
R Hargest
D Hargreaves
G Harper
N Harris
M Hawthorne
G Hellawell
S Hettiararchy
T Higginson
M Hilton
M Hilton
R Hind
T Holme
S Holtham
C Hopkins
J Hopkins
N Horlock
K Hosie
J Howell
J Huang
T Hui
D Hunt
M Hutton
C Ingham Clark
N Inston
F Iuwagwu
M Jackson
L James
N Jamieson
T Jefferis
B Jemec
G Johnson
O Jones
J Jones
S Joshi
B Jowett
P Kanabar
P Kang
R Kazi
R Kerry
A Khan
D Khoo
L King
A Kingsnorth
J Kitson
S Kohlhardt
R Kundra
D Lam
S lambert
P Lamont
I Lane
S Large
C Lavy
P Leach
I Learmonth
R Leicester
M Lewis
C Little
D Lobo
A Logan
D Loke
T Loosemore
B Lovett
M Lyons
R MacKenzie
S Madaan
D Mahon
A Majeed
S Mansfield
J Manson
N Markham
D Marsh
R Marshall
J Marshall
J Marshall
C Marx
W Mason
M McCarthy
I McDermott
M McFall
J McGrath
M McGurk
A McIrvine
R McKee
G Mead
D Menzies
K Metcalf
M Midwinter
C Milford
M Millwaters
Q Milner
A Mirnezami
D Mitchell
P Mitchell
T Mitchell
J Monson
M Moran
R Morgan
J Mosley
S Mostofi
S Mudan
M Mullan
T Mundy
C Munsch
M Murphy
G Myers
F Myint
G Nabi
A Najmaldin
S Nalagatla
D Nasmyth
P Nichols
M Nicholson
D Nielsen
P Nix
C Nnene
F Norman-Taylor
K Nugent
D Nunn
M Nuttall
T O Donnell
M Oddy
A Odurny
P O_Dwyer
O Oluwajobi
G Oniscu
F Pakzad
V Paleri
J Palmer
A Pandya
D Parikh
R Patel
N Patel
S Paterson-Brown
P Pavlou
V Peter
M Phillips
M Pickford
B Powell
J Press
R Pydisetty
J Pye
C Quick
J Quinlan
A Rahi
S Raimes
R Rajan
R Ramsden
S Rao
H Reece-Smith
D Rew
P Robb
D Roberts
T Rockall
A Roe
S Rogers
A Roposch
A Ross
P Sagar
V Sakthivel
P Sauve
R Savage
R Sayers
J Scholefield
P Schranz
H Scott
J Seaward
P Sedman
A Senapati
H Shaaban
M Shackcloth
J Shah
I Sharpe
E Shenouda
K Sherman
S Silverman
A Singh
B Singh
S Singh
A Sinha
M Sinha
G Sittampalam
J Skinner
D Skipper
F Smith
M Smith
P Smith
F Smith
E Sorene
J Southgate
L Spitz
R Squire
J Staiano
D Standley
D Stanley
M Stanton
I Stockley
C Stone
M Stott
I Tait
N Talbot
A Tavakkolizadeh
W Taylor
T Terry
A Thapur
T Theologis
P Thomas
J Thorfinn
C Thorn
R Tillman
M Timmons
A Toms
U Trivedi
R Turner
M Uglow
T Underwood
M Utukuri
D van Dellen
P Van Hille
S Varshney
P Vaughan-Shaw
V Vijay
R Vohra
S Wajed
C Wakefield
G Walker
A Walker
M Walsh
D Ward
J Warner
D Warwick
T Waters
L Watkins
A Watkinson
D Watkinson
A Watkinson
S Watson
A Watts
R Welbourn
R White
S White
G Williams
P Wilson
T Wilson
M Wilson
J Wilson-MacDonald
O Wiseman
C Wong
J Wong
C Woodhouse
T Wright
L Wyld
C Yiangou
C Yiu

